# Differential Regulation of Cell-Cell Contact, Invasion and Anoikis by hScrib and hDlg in Keratinocytes

**DOI:** 10.1371/journal.pone.0040279

**Published:** 2012-07-06

**Authors:** Paola Massimi, Patrizia Zori, Sally Roberts, Lawrence Banks

**Affiliations:** 1 International Centre For Genetic Engineering and Biotechnology Padriciano 99, Trieste, Italy; 2 Department of Cancer Sciences, University of Birmingham, Edgbaston, Birmingham, United Kingdom; University of Birmingham, United Kingdom

## Abstract

The components of the Scrib/Dlg tumour suppressor complex have complementary roles in Drosophila and loss of both proteins is a common event in many different human tumours. However no studies have directly addressed the respective contributions of loss of hScrib and hDlg in the same human cell background to cellular phenotypes associated with cell transformation. In human HaCaT keratinocytes we show that removal of hScrib greatly reduces cell-cell contact and cell-matrix interactions, and promotes an invasive phenotype. Conversely, in cells lacking hDlg1 cell-cell contacts are maintained and there are decreases in both cell growth and invasion. However, hDlg-depleted cells show increased resistance to a specialized form of apoptosis known as anoikis, to which cells lacking hScrib are highly susceptible. Thus whilst it has been widely assumed that hScrib and hDlg have complementary roles, these studies in fact demonstrate that hScrib and hDlg1 have distinct and opposing functions in human keratinocytes.

## Introduction

Control of cell polarity is a complex process involving the coordinate activity of three multi-molecular signaling complexes: the Crumbs complex, the Par complex and the Scrib complex [1], [2]. Through a series of antagonistic interactions the components of these three complexes control a number of downstream signaling complexes that contribute to the regulation of cell polarity and cell proliferation [3]. In many cases, the loss of different components of this pathway have been implicated in the development of human malignancies [1], [4], [5], [6], [7], and this has been borne out by studies in Drosophila and in mice [8], [9], [10].

The human hScrib complex consists of three proteins, hScrib, hDlg1 and Hugl-1. In Drosophila, loss of either Scrib or Dlg produces imaginal discs overgrowth and an invasive phenotype [3], [8]. In human cells, hScrib and hDlg1 appear to regulate important pathways governing cell polarity and cell attachment, and the mammalian equivalents can functionally complement loss of the corresponding protein in Drosophila [11], [12], [13]. There is also accumulating evidence that both proteins have potential tumour suppressor roles in the development of human malignancies. For example, loss of hDlg1 and hScrib appears to be a common feature in many late-stage epithelial tumours, including cervical, colon and breast cancers [5], [7], [14]. In addition, cervical cancer-causing Human Papillomaviruses (HPVs) can interact with, and inactivate, both hDlg1 and hScrib by the action of the E6 oncoprotein, further highlighting their potential tumour suppressive properties [15], [16].

More recent studies have begun to attempt to dissect the molecular mechanisms of action of hDlg1 and hScrib. In the case of hScrib, it appears to be a regulator of the JNK and ERK signaling cascades; loss of hScrib appears to contribute to mammary tumour development and to cooperate with the Ras and Myc oncogenes [17], [18], [19], [20]. Studies in Drosophila would also suggest highly interdependent functions for Dlg and Scrib, in that perturbation of one will also adversely affect the function of the other [8] although in human cells the hDlg1/hScrib interactions do not appear to be as simple [21], and loss of either hScrib or hDlg1 does not appear to unduly affect the pattern of expression of the other in human epithelial cells [21]. To date, detailed knockdown studies have only been performed on hScrib in MDCK and MCF10A [18], [22] cells, the latter being in the context of oncogenic Ras expression. However, no studies have been done to directly compare the effects of loss of hDlg1 and hScrib in either the same cell type or in human epithelial cells of squamous origin, where loss of either protein has been reported to occur during the course of human tumour development. To address this we have generated and characterized a series of keratinocyte lines lacking the hScrib and hDlg1 proteins. These studies define critical activities of each protein in the regulation of diverse aspects of cell survival, invasion, attachment and cell signaling.

## Results

### Perturbation of Epithelial Cell Morphology following hScrib Ablation

**Figure 1:**
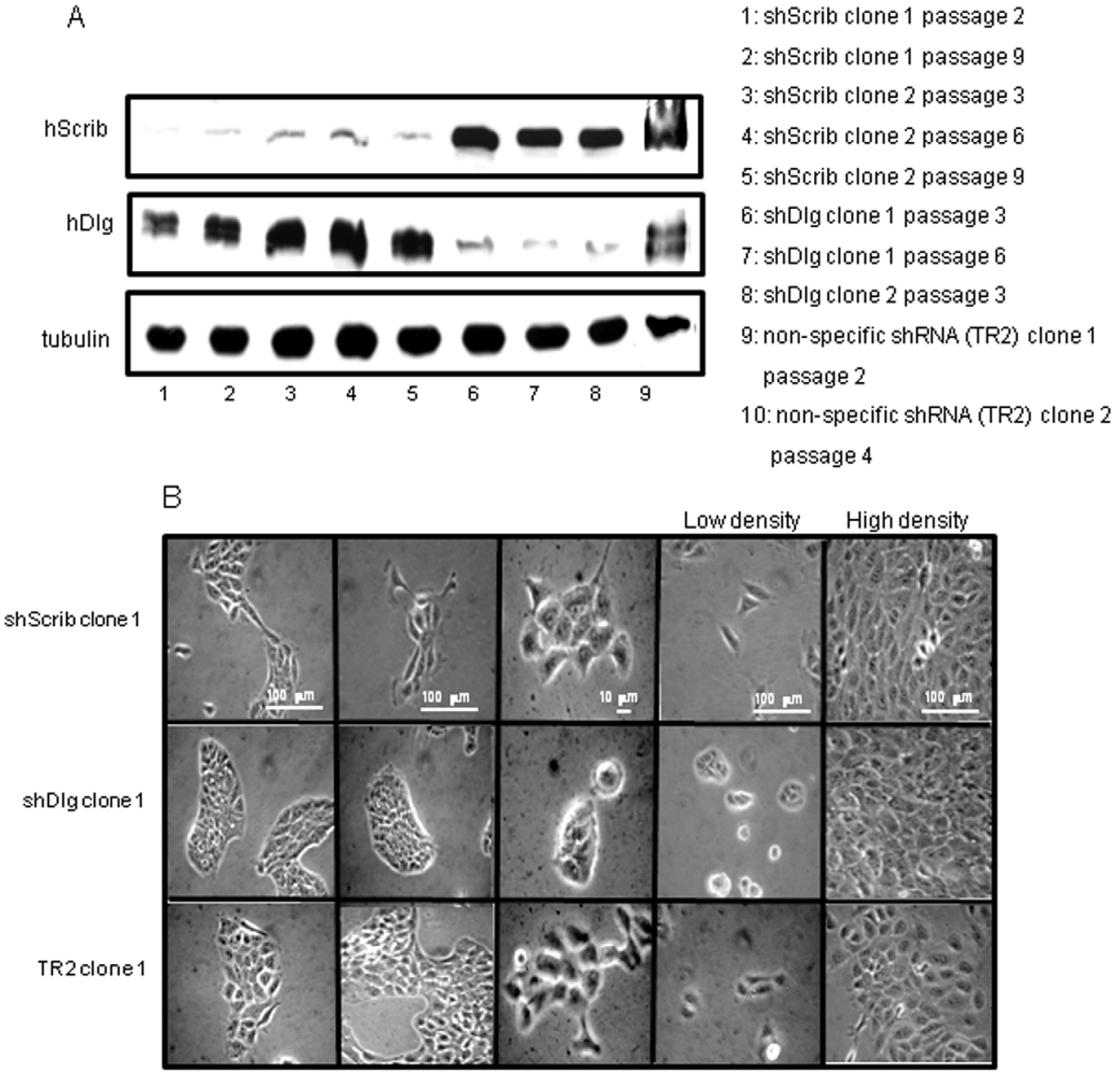
Generation of HaCaT cell lines stably depleted of hDlg1 or hScrib expression. Panel A. HaCaT cells were transfected with control plasmid (TR2) or plasmids with targeting sequences specific for either hDlg1 or hScrib. After selection, colonies were picked and levels of hDlg1 and hScrib expression were ascertained by western blotting. A selection of clones is shown at different passages, with the levels of tubulin expression shown for comparison. Panel B. Morphological appearance of the control (TR2 series) and representative hDlg1 and hScrib-depleted cell lines at low and high density. Note the typical HaCaT cobblestone morphology of the control cells and hDlg1-depleted cells, and the more fibroblastic appearance of cells in the hScrib-depleted clones at low cell densities. At higher cell density all three lines exhibit similar cobblestone morphology (right hand panels).

Loss of either Scrib or Dlg can have differing effects upon cellular homeostasis, depending upon the particular cellular context [8], [17], [18], [19], [23]. However there have been no studies to directly compare consequences of the loss of either protein in the same cell type and at the same time. Considering the potential context-dependent aspects to hDlg1 and hScrib function, we wanted to investigate the effects of the loss of hDlg1 and hScrib in human keratinocytes, which are the target cell for HPVs and in which the virus drives cell transformation and ultimately tumorigenesis; a process that is accompanied by loss of hDlg1/hScrib expression [6], [14]. To do this we used HaCaT cells, a non-tumourigenic keratinocyte cell line derived from adult trunk skin [24], that were stably transfected with commercial shRNA targeting vectors directed against hScrib and hDlg1. The resulting clones were analysed for the levels of hDlg1 and hScrib expression by western blotting. The results in Figure 1A show that several stable cell lines were obtained with reduced levels of either hDlg1 or hScrib when compared with the control clones transfected with a vector coding for non-specific scrambled shRNA.

The general morphology of the cells at low and high cell densities was also monitored. As can be seen from Figure 1B, a number of marked differences are apparent at low cell density. The hDlg1 knockdown cells grow in very tight clusters and the clones maintain the cobblestone morphology apparent in cultures of the control cells. In contrast, the hScrib knockdown cells have lost the cobblestone morphology and cells are more elongated and have a fibroblast–like appearance. However at high cell densities all three lines display similar cobblestone morphology.

**Figure 2:**
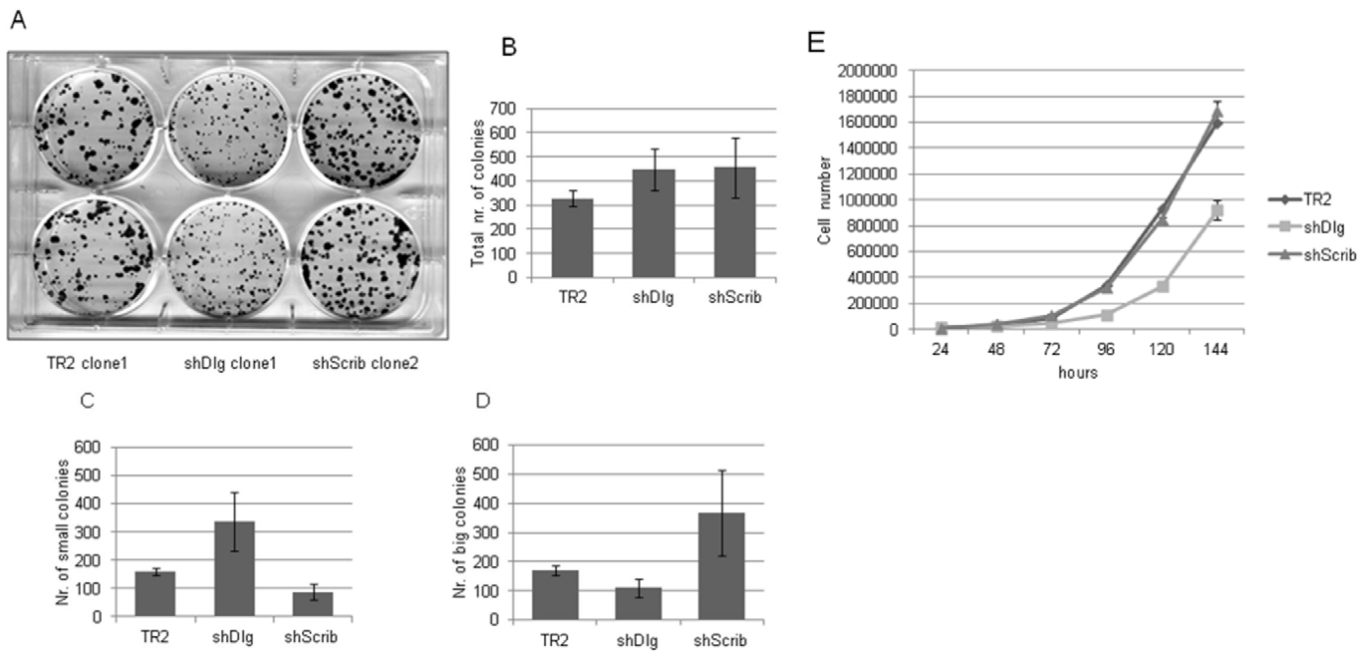
Comparison of the colony-forming capacity of the different cell lines. 1×10^3^ cells were plated in each well and after 10 days the dishes were fixed and stained with Giemsa. Panel A shows a representative assay with the indicated clones. Panels B–D show the collated results from a minimum of 3 assays with at least two different clones. Panel B shows the total colony numbers, with this separated into the number of small (Panel C) and large colonies (Panel D) based on the pixel cell density (where small colonies are <0.012 mm^2^ and large >0.012 mm^2^) of the image using a BioRAD Gel Doc xR. Students T Test on Panel C: TR2 vs Dlg p = 0.0171, TR2 vs Scrib p = 0.0201 and on Panel D: TR2 vs Dlg p = 0.0569, TR2 vs Scrib p = 0.0381. Panel E shows the growth rates of representative clones of the TR2, shScrib and shDlg1 cell lines over a period of 6 days.

We also analysed the capacity of the cells to form colonies. The results shown in Figure 2 demonstrate similar colony-forming capacity between all the cell lines (Figures 2A and 2B), although the rates of colony growth were consistently and statistically significantly slower in the hDlg1 depleted cells (Figures 2A and 2C showing small colonies as opposed to large colonies in Figure 2D). In contrast, a small but consistent and statistically significant increase in colony size and number was obtained with all the hScrib depleted cell lines (Figures 2A and 2D). To determine whether there was any intrinsic difference in the growth rates of the different cell lines the cells were counted over a number of days and the results obtained are shown in Figure 2E. As can be seen, the hDlg1 depleted cells also exhibit somewhat slower rates of proliferation when compared to the control and hScrib depleted cell lines.

These studies demonstrate that loss of either hDlg1 or hScrib has distinct effects with respect to cell morphology and colony growth.

### Loss of hScrib and hDlg1 have Different Effects upon Cell-cell Contact

**Figure 3:**
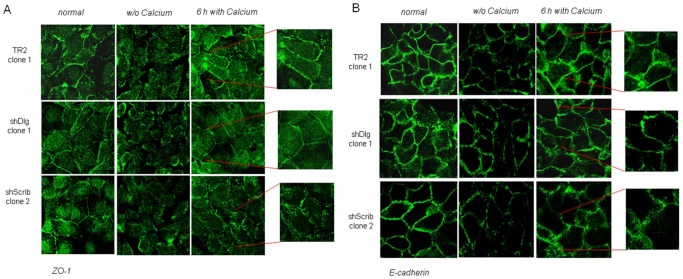
hScrib-depleted cells show reduced TJ and AJ formation. Control, hDlg1 and hScrib-depleted cells were grown for 24 h then switched into low calcium medium for 1 h. High calcium containing medium was then added for 6 h. The cells were fixed and stained for the TJ-associated marker ZO-1 (Panel A with insets showing junctional detail) and the AJ-associated marker E-cadherin (Panel B with insets showing junctional detail). The results show the staining under normal growth conditions, after calcium starvation and after 6 h calcium addition in representative clones; identical results were obtained with at least two additional clones of control, shRNA Dlg1 and shRNA Scrib cells.

Previous studies have implicated a role for the hScrib complex in the formation of tight junctions (TJs) and adherens junctions (AJs) [22], [25]. Therefore we proceeded to analyse the capacity of the hDlg1 and hScrib-ablated cells to re-form TJs and AJs following calcium depletion, using ZO-1 as a marker for TJ assembly and E-cadherin as a marker for AJ assembly. Cells were placed in a low concentration of calcium for 1 h, and then switched back to a higher concentration for up to 6 h. The restoration of TJs and AJs was determined by immunofluorescence analysis. The results demonstrate that the hScrib-depleted cells are impaired in their ability to reform both TJs and AJs, with a significant delay in ZO-1 (Figure 3A) and E-cadherin (Figure 3B) accumulation compared with the control cells, and this is in agreement with previous studies [22], [25]. In contrast, the hDlg1-depleted cells appear to re-establish TJs and AJs in a manner similar to the control cells.

**Figure 4:**
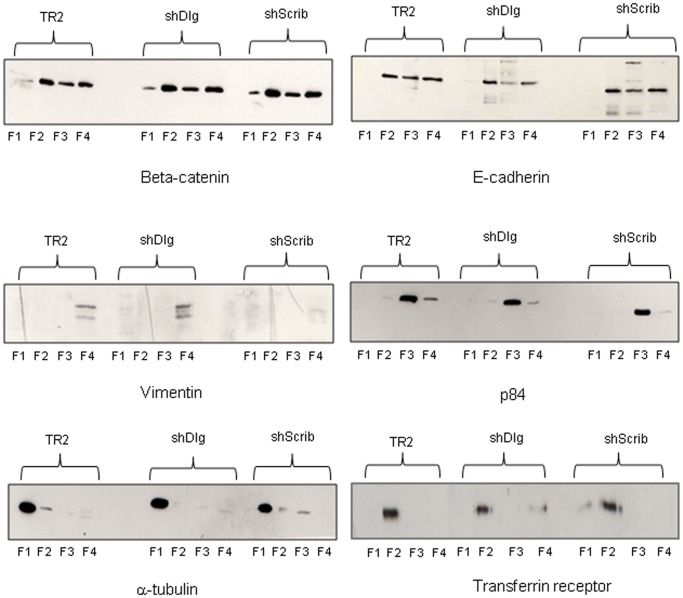
hDlg1 depleted cells show reduced levels of E-cadherin expression but increased levels of β-catenin expression. Cells were grown to 80% confluency and then extracted into cytosolic (F1), membrane (F2), nuclear (F3) and cytoskeletal (F4) fractions. These were then analysed for the distribution of β-catenin and E-cadherin by western blotting. Controls for the integrity of the fractions were vimentin for the cytoskeletal fraction, p84 for the nuclear fraction, α-tubulin for the cytosolic fraction and transferrin receptor for the membrane fraction.

We also compared the relative levels of expression and subcellular distribution of E-cadherin and β-catenin in the hDlg1 and hScrib depleted cell lines. The cells were grown to 80% confluency and seperated into cytosolic, membrane, nuclear and cytoskeletal fractions and the pattern of β-catenin and E-cadherin expression monitored by western blotting. The results in Figure 4 show a modest 50% increase in the levels of β-catenin in the membrane fraction of the hDlg1 depleted cells and a 114% increase in β-catenin levels in the cytoplasmic fraction of the hScrib depleted cells, in comparison to the control cells. Interestingly, the levels of E-cadherin are reduced by 40% in the cytoplasmic fraction in the hDlg1 depleted cells, but are increased in the same fraction by 150% in the hScrib depleted cells.

**Figure 5:**
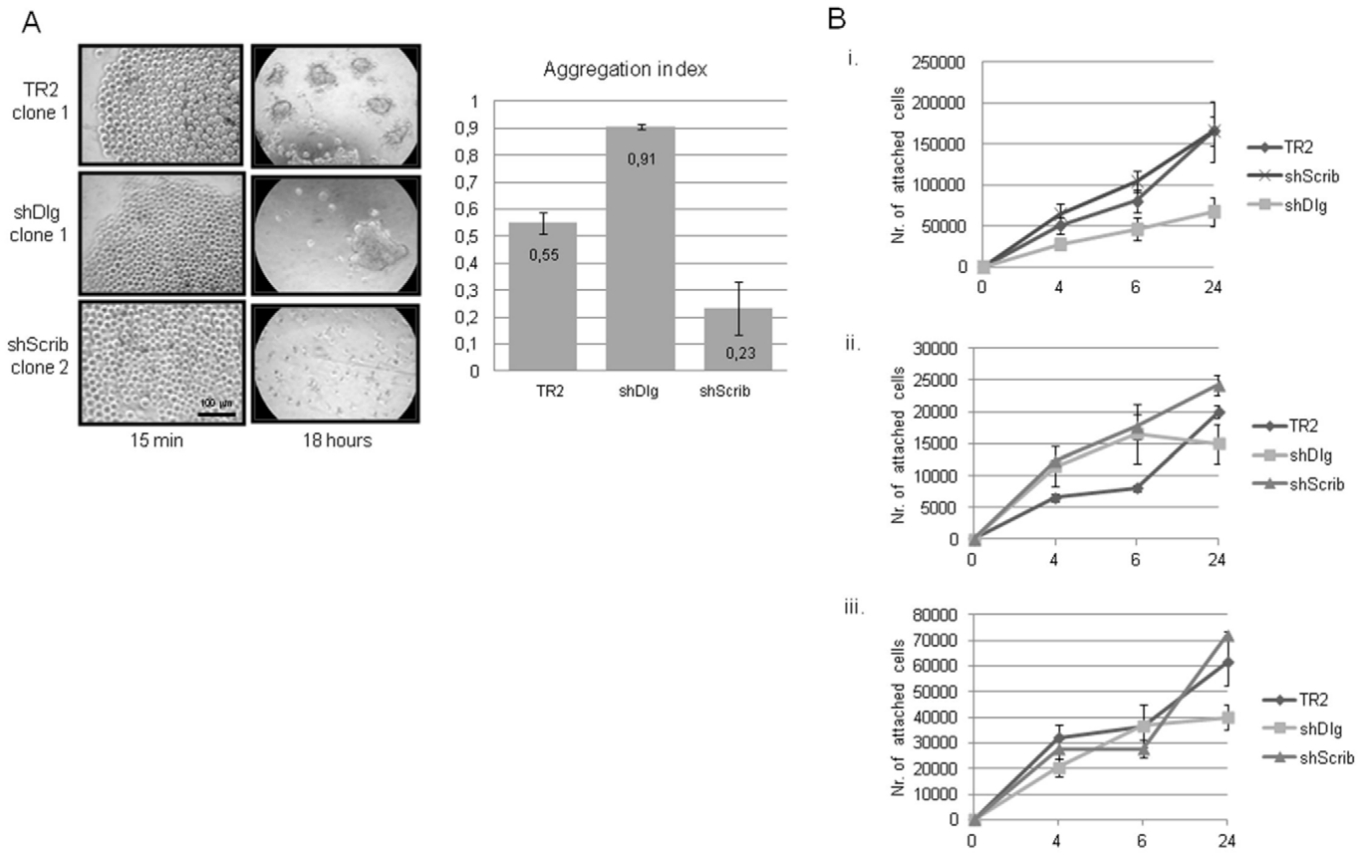
hScrib depleted cells exhibit reduced levels of cell-cell contact. Panel A. Cells were suspended from a plastic tissue culture dish lid in a drop of medium and incubated overnight. The cells were photographed after 15 mins and after 18 h incubation in suspension, where a single cell suspension can be seen at the 15 mins time point and aggregates of cells at the 18 h time point. The graph shows the aggregation index calculated from at least three independent experiments using two independent control, hDlg1 and hScrib depleted cell lines. Error bars are also shown. Panel B. Cells were plated on plastic tissue culture dishes that were either untreated (i), coated with fibronectin (ii) or coated with collagen (iii) and the attached cells counted at different time points. The graphs show the results from at least three independent assays using three independent control, hDlg1 and hScrib depleted cell lines and the standard deviations are shown.

As an alternative measure of the capacity of the cells to form cell-cell contacts, we assessed the capacity of the cells to adhere to one another in a hanging-drop assay. Cells were placed in a drop of medium and inverted on a plastic tissue culture dish lid and left overnight at 37°C. After this time the cells were visualized, the numbers of cell clumps counted and the aggregation index determined. The results in Figure 5A show that whilst hScrib-depleted cells have a lower aggregation index (0.23) than the control cells (0.55), the aggregation index of hDlg1-depleted cells is higher (0.91), with very large clumps of cells present after the overnight incubation.

We also monitored the capacity of these cells to establish substratum interactions. Cells were plated on plastic tissue culture dishes that were either untreated or coated with collagen or fibronectin, and the attached cells were counted over a period of hours. The results in Figure 5B show that on untreated dishes the control and hScrib-depleted cells attach with similar kinetics, whilst the hDlg1 depleted cells attach with slower kinetics. In contrast, on fibronectin treated dishes hDlg1 and hScrib depleted cells attach similarly and faster than control cells, whereas on collagen treated dishes all three lines attach with similar efficiency. Taken together, these results demonstrate that the loss of hScrib significantly decreases the capacity of keratinocytes to form stable cell-cell contacts and re-form TJs, but appears to have little affect on cell substratum interactions. In contrast, in the same assay systems, loss of hDlg1 whilst apparently enhancing the capacity of the cells to adhere to each other in a hanging drop assay, at the same time compromises the capacity of the cells to adhere in a manner that is dependent upon the particular substratum.

### hScrib-depleted Cells Exhibit Increased Invasiveness

**Figure 6:**
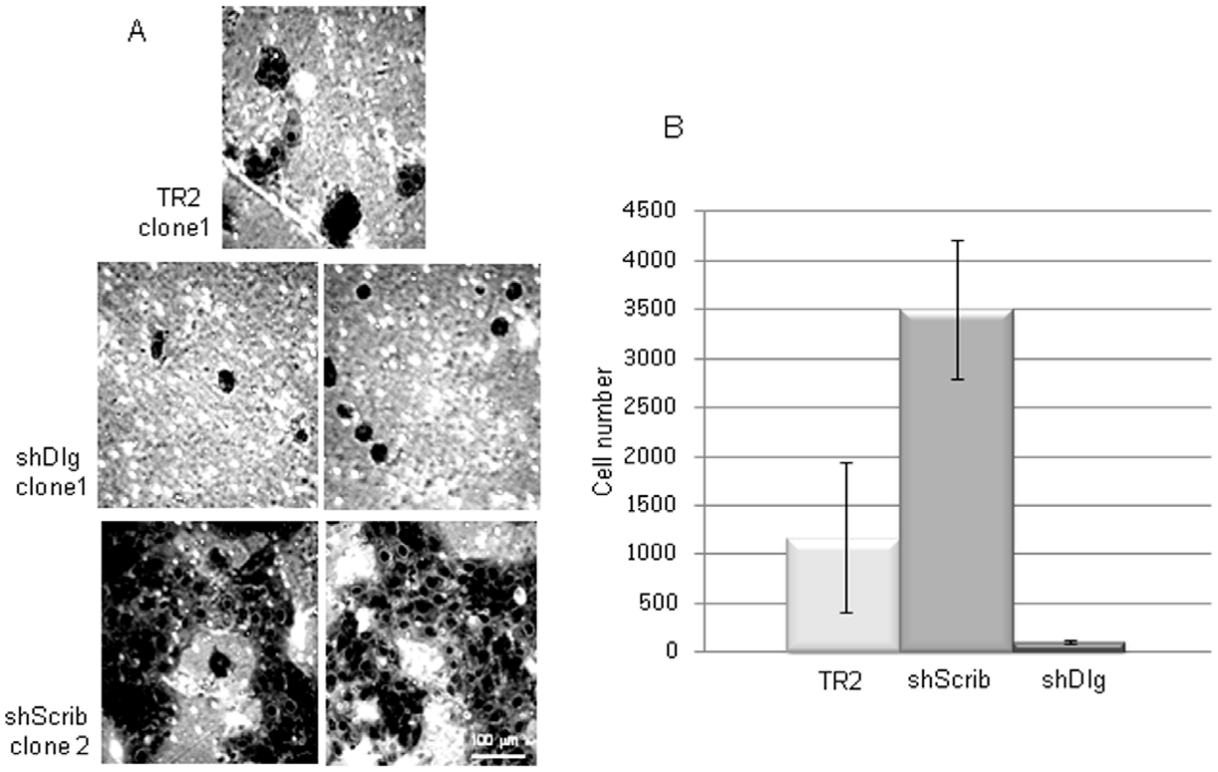
Loss of hScrib enhances cell invasion. Panel A. The cell lines were incubated in the upper compartment of matrigel chambers in serum-free medium. After 22 h, the cells that had migrated into the lower serum-containing compartment were fixed and stained with Crystal Violet. The panels show the staining of the cells on the lower surface of the collagen matrix, with the TR2 control cell line and two examples of hDlg1 depleted cells and two examples of the hScrib-depleted cells shown. Panel B shows the quantifications and standard deviations of the numbers of invading cells obtained from at least three independent assays with at least three different control, hScrib and hDlg1 knockdown cell lines.

Previous studies have shown that loss of Scribble can enhance the invasive capacity of cells in the presence of an activated Ras or Myc oncogene [17], [18], whilst there are no reports on the effects of hDlg1 loss upon a cell’s invasive capacity. To determine whether loss of hScrib or hDlg1 in the absence of oncogene activation in keratinocytes can affect the invasive capacity of the cells, we performed a series of matrigel invasion assays. Cells were plated on matrigel chambers in serum-free medium and their capacity to migrate to the lower serum-containing chamber was assessed after 22 h. The results in Figure 6A and 6B, show that loss of hScrib alone increases the invasive capacity of the keratinocytes by over 3 fold compared to control cells. Interestingly, loss of hDlg1 appears to decrease the invasive capacity of the keratinocytes by almost 10 fold, with far fewer cells migrating through the matrix in comparison with the control cells.

As an additional verification of these studies we extended the time of the invasion assays to 48 h, and obtained similar results (Figure S1). We also counted the number of cells attaching to the upper surface of the matrigel chamber after 1 h and after an overnight incubation. As can be seen from Figure S2 similar numbers of cells had attached at both time points. This also allowed us to determine the total percentage of cells migrating through the matrigel over the course of the assay and this is also shown in Figure S2. To exclude the possibility that invading hDlg1 depleted cells had become detached from the lower surface of the matrigel chamber, the medium was harvested and analysed for any floating cells: none were detected in any of the analyses. Finally, to exclude any off target effects of the targeting vectors, invasion assays were done following transient siRNA ablation of either hScrib or hDlg1. In both cases, similar results were obtained, with loss of hScrib increasing cell invasion (data not shown) and loss of hDlg1 reducing cell invasion [26]. Taken together these results demonstrate that hScrib depleted cells have a significantly increased invasive potential whilst hDlg1 depleted cells have a decreased capacity to invade.

Having found that hScrib depleted cells had increased invasive potential we also ascertained whether the hScrib and hDlg1 depleted cells were capable of growing in an achorage independent manner. Cells were suspended in 0.5% soft agar and allowed to form colonies over a period of 10 days. After this time the percentage of cells forming colonies was ascertained and the results obtained are shown in Figure S3. As can be seen, only the hScrib depleted cells show any significant ability to grow in soft agar, albeit to only a low level.

### Loss of hDlg Enhances Resistance to Anoikis

**Figure 7:**
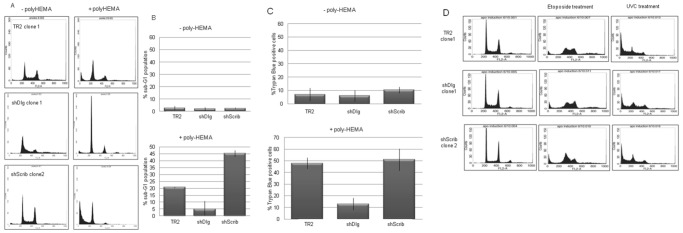
Loss of hDlg1 enhances resistance to anoikis. Panel A. Cells were plated on tissue culture dishes that were either untreated, or coated with poly-HEMA to block cell attachment. After 24 h the cells were harvested and stained with Propidium Iodide, and the cell cycle distribution of a representative clone was ascertained by flow cytometry. Panel B shows the mean percentage of cells entering apoptosis (as determined by sub-G1 DNA content) from at least three separate experiments with three independent cell lines together with the standard deviations. Panel C. Cells were processed as in Panel A, but were stained with Trypan Blue. The results show the mean percentage of dead and dying cells, as determined by Trypan Blue uptake from at least three independent experiments on at least three independent cell lines together with the standard deviations. Panel D. Cells were treated with etoposide or exposed to UV C. After 12 h incubation the cells were stained with Propidium Iodide and the sub-genomic DNA content determined by flow cytometry, with the results shown being from one representative clone of each line.

Previous studies have implicated Scrib in the regulation of apoptosis, with the protein exerting either pro- or anti-apoptotic effects depending upon the particular experimental setting, frequently in the context of oncogene activation [17], [18], [27], [28]. In contrast, there are no reports on any potential roles of hDlg1 in the regulation of apoptosis. A major response of cells to loss of cell-substratum interaction is to enter the apoptotic program of anoikis [29], and we were therefore interested in determining how the hDlg1 and hScrib-knockdown cells would respond to such an apoptotic stimulus. To do this, cells were plated on poly-HEMA coated dishes for 24 h, such that they cannot form a cell-substratum interaction. After 24 h the cells were harvested and levels of apoptosis were determined by propidium iodide staining and flow cytometry. The results shown in Figure 7A and 7B demonstrate that 20% of the control cells have entered apoptosis after 24 h in a substratum-free environment, as determined by the proportion of cells with a sub-G1 DNA content. Interestingly, this proportion is somewhat greater (40%) in the hScrib-depleted cells, supporting those studies indicating an anti-apoptotic function for the protein [17], [28]. In contrast, the hDlg 1-depleted cells appear to be highly resistant to anoikis, with only a small fraction (5%) of the cells showing sub-G1 DNA content. As a further measure of apoptotic potential, we also stained the cells with trypan blue to detect dead and dying cells. The results obtained in Figure 7C show that almost 50% of the cells are dead or dying on poly-HEMA treated dishes in both the control and hScrib-depleted cell lines. In contrast there are many fewer trypan blue staining cells (reduced by almost 5 fold to 10%) in the hDlg1-depleted population of cells. We also analysed cells in which rat Dlg was re-expressed in the hDlg1 depleted cells (Krishna Subbaiah et al., 2012), and as can be seen from Figure S4 these cells regain sensitivity to anoikis induction. Taken together, these results indicate that loss of hDlg1 can enhance the resistance of a cell to anoikis.

We next wanted to determine whether loss of hDlg1 could enhance the resistance of keratinocytes to a broad range of apoptotic stimuli. To investigate this we analysed the susceptibility of the different cell lines to undergo apoptosis following DNA damage induced by etoposide for 12 h or following exposure to a short burst of UV-C irradiation and then again cultured for a further 12 h. Cells were harvested and the sub-genomic DNA was determined by propidium iodide staining and flow cytometry. The results in Figure 7D show similar levels of apoptosis in response to both treatments in the control cells, and in the hDlg1 and hScrib-depleted cells. These results demonstrate that the enhanced resistance to anoikis observed upon hDlg1 knockdown is not due to a generalized overall resistance of the cells to enter apoptosis, but rather is highly specific to anoikis-inducing stimuli.

## Discussion

The cell polarity complex comprising Scrib, Dlg and Lgl is essential for regulating cell polarity and proliferation in Drosophila, with loss of any component resulting in broadly similar and complementary phenotypes. In human cells the functions of these proteins are less clear, although tumour suppressor activity has been proposed directly for hScrib [17], [18], [20], and implied for hDlg1 [4]. Furthermore, both hDlg and hScrib can complement the loss of their respective homologue in Drosophila [11], [12], suggesting a degree of conservation of function across evolution. In this study we show that in human keratinocytes, hDlg1 and hScrib contribute to very different aspects of cellular homeostasis, with loss of either producing marked differences in the ability of cells to form contacts, form colonies, invade and enter apoptosis.

Based on these studies, we provide strong evidence of a role in tumour suppression for hScrib. Depletion of the protein results in a loss of the epithelial cobblestone morphology at low cell density, with the cells adopting a more fibroblast–like morphology, although at high cell densities the cells have a cobblestone epithelial morphology. We also find that loss of hScrib is sufficient to significantly increase the invasive potential of the keratinocytes by over 3 fold in comparison to the control cells. Thus whilst previous studies have shown that loss of hScrib can do this in the presence of an activated ras oncogene [18] our studies demonstrate that loss of hScrib can enhance cell invasion without the addition of an exogenous oncogene. In addition hScrib depleted cells also display weak growth in soft agar. Taken together these results demonstrate a potent tumour suppressor activity for hScrib.

In keratinocytes, loss of hDlg1 has some important consequences, many of which are unexpected. Previous studies in Drosophila have implicated Dlg as being important for the regulation of cell-cell contact [30]. However, loss of hDlg1 in keratinocytes appears to have little effect on TJ and AJ formation in comparison to the control cells. Hanging drop assays actually demonstrated an increased propensity of the hDlg1-depleted cells to aggregate, when compared with the control and hScrib-depleted cells. Thus loss of hDlg1 in keratinocytes does not seem to unduly perturb the capacity of the cells to form contacts. It should be stressed that this is not a reflection of the assay systems, since the hScrib-depleted cells behaved exactly as would be expected from previous studies, showing reduced levels of cell-cell contact and a delay in TJ and AJ re-formation [22], [25].

Keratinocytes lacking hDlg1 also exhibit slower rates of cell proliferation and reduced rates of cell attachment, both features that most likely contribute to reduced rates of colony growth. Furthermore we also found that loss of hDlg1 greatly decreased invasive potential; this is in agreement with our recent studies on the role of hDlg1 in HPV induced malignancy, where this activity of hDlg1 appears to be mediated through its capacity to regulate RhoG activity and subsequent epithelial cell movement and invasion [26]. Based on these phenotypes, one could propose that hDlg1 has a degree of proto-oncogenic potential. Interestingly, this is in agreement with previous studies which demonstrated that certain isoforms of hDlg1 in the presence of Adenovirus 9 E4-ORF1 also possess oncogenic activity [31].

The most intriguing phenotype obtained through loss of hDlg1 was the increased resistance of the cells to undergo anoikis. Whilst previous studies had implicated hScrib in the regulation of diverse apoptotic pathways [17], [18], [27], [28], little is known about hDlg1. We first chose to analyse anoikis, as there was no information on any potential role for either hDlg1 or hScrib in the control of this particular apoptotic response. Upon plating cells onto poly-HEMA treated tissue culture dishes, such that no cell-substratum attachment was possible, the control cells demonstrated high levels of sub-genomic DNA, indicative of entry into apoptosis. Interestingly, the hScrib-depleted cells appeared even more susceptible to this particular form of apoptotic stimulus, supporting previous studies suggesting that hScrib exerts an anti-apoptotic response in certain cell contexts [17], [28]. In contrast, the hDlg1-depleted cells showed marked resistance to anoikis. These results were also confirmed by the use of a viable stain trypan blue to detect non-viable cells, demonstrating a high level of cell death in the control and hScrib-depleted cells, whereas the hDlg1-depleted cells were largely unaffected. These results demonstrate that, in the context of anoikis, hDlg1 has a potent tumour suppressor potential.

Loss of expression of both hDlg1 and hScrib are frequent events in the later stages of tumourigenesis [5], [7], [14], although in earlier stages of disease progression, the levels of hScrib and hDlg1 expression can be extremely high, albeit often mislocalised [14], [32]. How these changes in the levels and patterns of hDlg1 and hScrib expression contribute to carcinogenesis, and whether they are in any way cooperative, still remains to be determined. However based on the above studies, one can readily envisage a scenario where the primary effects of loss of hDlg1 are reduced rates of proliferation and enhanced resistance to anoikis, characteristics that are common to many types of tumour. Loss of hScrib on the other hand may well contribute to the invasive potential of the tumour cell. Therefore cooperative effects in loss of hScrib and hDlg1 in driving tumour progression is quite possible, and in the case of HPV-induced cervical cancer, loss of either protein could be expected to have dramatic consequences for the capacity of the cells to invade and survive. It is intriguing to speculate that HPV E6, by differentially targeting hScrib and hDlg1, may fine-tune the levels of both proteins, such that optimal outcomes for viral replication or cancer progression, will be attained.

## Materials and Methods

### Cells and their Characterization

HaCaT (human keratinocytes) cells [24] were cultured in DMEM supplemented with 10% fetal bovine serum, penicillin–streptomycin (100 U/ml) and glutamine (300 µg/ml) in a humidified 5% CO_2_ incubator. To generate the depleted hDlg1 and hScrib cell lines, HaCaT cells were transfected with a pool of short hairpin RNA constructs (Euroclone) against hScrib and hDlg1, using Lipofectamine 2000 (Invitrogen, Milan, Italy) according to the manufacturer’s protocol. The cells were selected with puromycin (500 ng/ml) and after 4 weeks single colonies were analysed for hScrib and hDlg1 expression by western blotting. At least three separate clones of each were used in this analysis. Parallel transfections and selections were performed using empty vector to generate control clones (TR) that had been subjected to the drug selection. Rescue clones of the hDlg1 KD cells were selcted following transfection with targeting vector resistant rat Dlg and a neomycin resistance marker.

### Antibodies

The following commercial antibodies were used at the dilution indicated: anti-hScrib goat polyclonal antibody (Santa Cruz, CA, USA; Western Blot 1∶1000), anti-hDlg1 mouse monoclonal antibody (Santa Cruz, CA, USA; Western Blot 1∶1000), anti-γ-tubulin mouse monoclonal antibody (Abcam; Western Blot 1∶5000), anti-ZO-1 rabbit polyclonal antibody (Santa Cruz; CA,USA; Immuofluorescence 1∶100), anti β-catenin mouse monoclonal (Santa Cruz; CA, USA; Western Blot 1∶500), anti E-Cadherin rabbit polyclonal (Santa Cruz; CA, USA; Western Blot 1∶500), anti- Vimentin mouse monoclonal (Santa Cruz; CA, USA; Western Blot 1∶1000), anti-p84 mouse monoclonal (Santa Cruz; CA, USA; Western Blot 1∶1000), anti-transferrin receptor mouse monoclonal (Santa Cruz, USA; Wesern Blot 1∶1000).

### Immunofluorescence and Microscopy

Cells were grown on glass coverslips and fixed in 3.7% paraformaldehyde in phosphate-buffered saline (PBS) for 20 mins at room temperature and processed as described previously [21].

### Western Blotting

Preparation of cell extracts and subsequent processing for western blotting were done as described previously [21].

### Collagen and Fibronectin Coating

CollagenType I derived from rat tail (BD #354236) was used to coat the dishes for 1 h at room temperature at a concentration of 1 mg/ml in PBS. The dishes were washed three times with PBS and then used as normal. For the fibronectin coating (F0895; Sigma stock solution 1 mg/ml), fibronectin was diluted to 1 µg/ml in PBS and applied to the dishes for 1 h at room temperature. The dishes were washed three times with PBS and then used as normal.

### Subcellular Fractionation Assay

Cells were plated at 40% density and after 24 h, differential extraction was performed to obtain cytosolic, membrane, nuclear and cytoskeletal fractions using the ProteoExtract Fractionation Kit (Calbiochem) according to the manufacturer’s instructions.

### Colony Forming Assays

The different cell lines were seeded in 6 well tissue culture dishes at the same concentration (1×10^3^) and grown for 10 days. Cells were then fixed with 10% paraformaldehyde for 20 mins and stained with Giemsa blue 10% in PBS for 20 mins. Pictures of the blue colonies were taken with Coolpix Nikon 995 Camera and quantified using BioRad Gel Doc™ XR.

### Calcium Switch Assays and TJ/AJ Assembly

Disassembly and restoration of TJs and AJs was carried out as described previously [33]. The HaCaT shRNA cell lines were seeded on coverslips and after 24 h were incubated for 1 h at 37°C in 2 mM EDTA in PBS to chelate calcium. Cells were then washed and either fixed immediately or incubated at 37°C in complete media for 6 h before fixation and IF analysis.

### Aggregation Assays

Cells were trypsinised in the presence of EDTA, washed twice in PBS, and resuspended at a concentration of 2.5×10^5^ cells/ml in DMEM/10% FBS. Drops (20 µl each) of medium, containing 5×10^3^ cells/drop, were pipetted onto the inner surface of a dish lid placed on the Petri so that the drop was hanging from the lid with the cells suspended within it. To avoid evaporation, 8 ml of serum-free culture medium were placed in the bottom of the Petri dish. After incubation overnight at 37°C, the lid of the Petri dish was inverted and the cells were photographed using an inverted tissue culture microscope at X40 magnification with a Coolpix Nikon 995 Camera.

The extent of cell aggregation is represented by the index [No-Nt]/No where Nt is the total particle number after the incubation time, t, and No is the total particle number at the initiation of incubation.

### Matrigel Invasion Assays

For the invasion assays we used a modified Boyden Chamber (Becton Dickinson), brought to room temperature and rehydrated with DMEM without serum. Cells were seeded at a concentration of 1×10^5^ cells in 200 µl of growth medium into the upper chamber. After allowing the cells to attach for 1 h, the medium in the upper chamber was replaced with DMEM without serum while DMEM/10% serum was added to the lower chamber as chemoattractant. After 22 h media and any cells remaining in the upper chamber were removed by wiping with a cotton swab. Cells that had invaded the lower chamber were fixed and stained with 0.5% Crystal Violet in 5% glutaraldehyde for 10 mins. They were then washed and examined using a transmitted light microscope at 20X magnification. At least three fields per membrane were counted for each cell line.

### Soft Agar Assays

For growth in soft agar cells were resuspended in growth medium containing 0.5% agar and then plated onto 6 cm tissue culture dishes. After 10 days incubation the percentage of cells forming colonies were counted.

### Apoptosis Assays

Tissue culture plates were coated with poly-HEMA (Sigma) by adding 5 ml of a 20 mg/ml poly-HEMA/95% ethanol solution to each 10 cm plate. After drying the plates for 2 h, cells were plated at 2.5×10^5^ cells/ml on either the poly-HEMA or untreated tissue culture plates and then incubated at 37°C for 24 h before harvesting. DNA damage induced apoptosis was caused by overnight treatment with etoposide (10 µM) or by a 10 sec exposure to UVC followed by a 12 h incubation.

### Propidium Iodide Staining and FACS Analyses

Cells were trypsinised and washed with PBS. The pellet was resuspended in Propidium Iodide staining solution [3.8 mM sodium citrate, 50 mg/ml PI, (Sigma P4170) 10 mg/ml RNAse A (Boeringher Mannheim), 10% NP40 in PBS] and incubated at 37°C in the dark for 20 mins. The cells were subjected to FACS analysis using a FACScalibur Cell Sorter (Becton Dickinson).

### Trypan Blue Staining

Cells were trypsinised and washed with PBS and then resuspended in a solution of 50% Trypan Blue 0.4% (Sigma)/PBS and counted with a transmitted light microscope at 20X magnification.

## Supporting Information

Figure S1 **Loss of hScrib enhances cell invasion.** The cell lines were incubated in the upper compartment of matrigel chambers in serum-free medium. After 48 h, the cells that had migrated into the lower serum-containing compartment were fixed and stained with Crystal Violet. The graph shows the mean number of invading cells obtained from two independent assays with a clone of control, hScrib and hDlg1 knockdown cell lines.
(TIF)

Figure S2 **Loss of hScrib or hDlg1 does not perturb attachment to the matrigel chambers.** The cell lines were added to the matrigel chambers and after 1 h and 22 h the numbers of cells attached to the upper chamber were counted. The upper two panels show the numbers of cells attached at the two time points and are the mean of two independent assays. The percentage of cells migrating through the matrigel was calculated based on the numbers that had attached and this is shown in the lower panel. Numbers are the mean from two independent experiments.
(TIF)

Figure S3 **Loss of hScrib confers weak growth in soft agar.** Cells were resuspended in medium containing 0.5% agar and allowed to grow for 10 days. The graph shows the numbers of colonies as a percentage of the total number of cells. Note the very weak increase in the capacity of the hScrib depleted cells to grow in soft agar.
(TIF)

Figure S4 **Restoration of Dlg expression restores sensitivity to anoikis.** hDlg1 depleted cells and cells re-expressing rat Dlg (Panel A) were plated on poly-HEMA coated dishes. After 24 h the cells were harvested and stained with Propidium Iodide, and the cell cycle distribution of two representative clones was ascertained by flow cytometry.
(TIF)
