# Supplementary figures and images for: Differential Regulation of Cell-Cell Contact, Invasion and Anoikis by hScrib and hDlg in Keratinocytes
Source: PLoS One. 2012 Jul 6;7(7):e40279. doi: 10.1371/journal.pone.0040279 (PMC3391271; doi:10.1371/journal.pone.0040279)

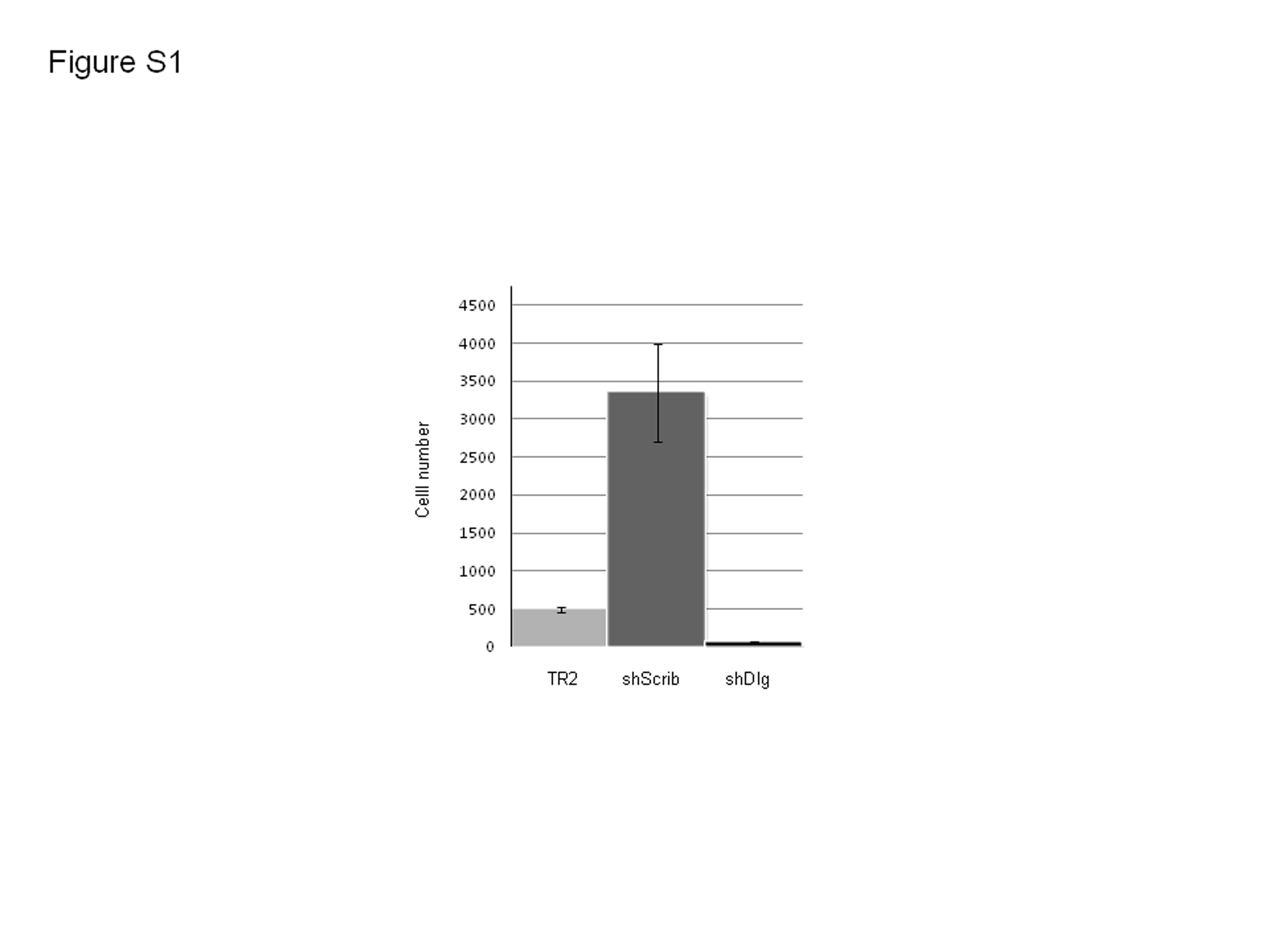

Supplement: Figure S1 — Loss of hScrib enhances cell invasion. The cell lines were incubated in the upper compartment of matrigel chambers in serum-free medium. After 48 h, the cells that had migrated into the lower serum-containing compartment were fixed and stained with Crystal Violet. The graph shows the mean number of invading cells obtained from two independent assays with a clone of control, hScrib and hDlg1 knockdown cell lines. (TIF) [file pone.0040279.s001.tif]

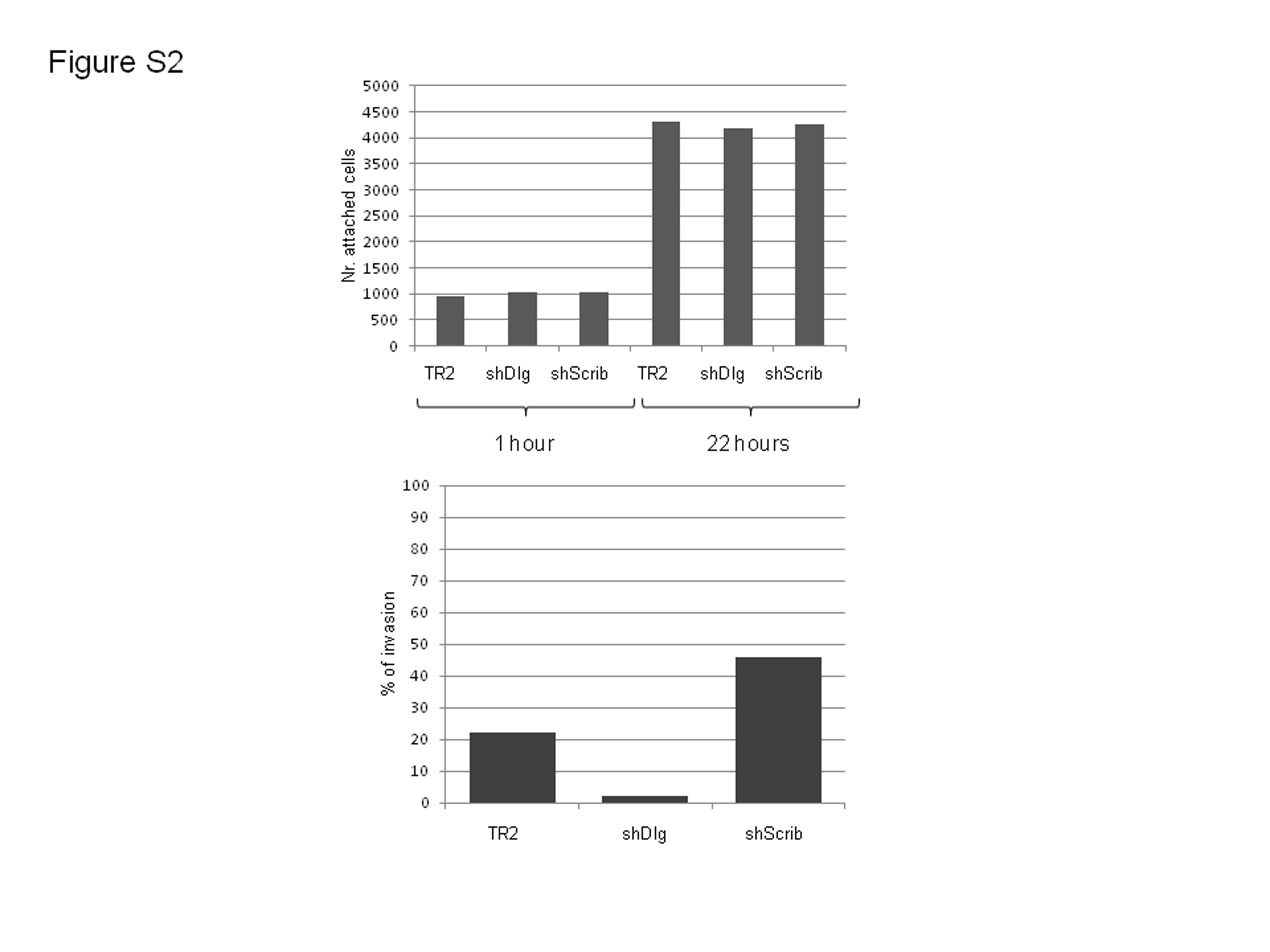

Supplement: Figure S2 — Loss of hScrib or hDlg1 does not perturb attachment to the matrigel chambers. The cell lines were added to the matrigel chambers and after 1 h and 22 h the numbers of cells attached to the upper chamber were counted. The upper two panels show the numbers of cells attached at the two time points and are the mean of two independent assays. The percentage of cells migrating through the matrigel was calculated based on the numbers that had attached and this is shown in the lower panel. Numbers are the mean from two independent experiments. (TIF) [file pone.0040279.s002.tif]

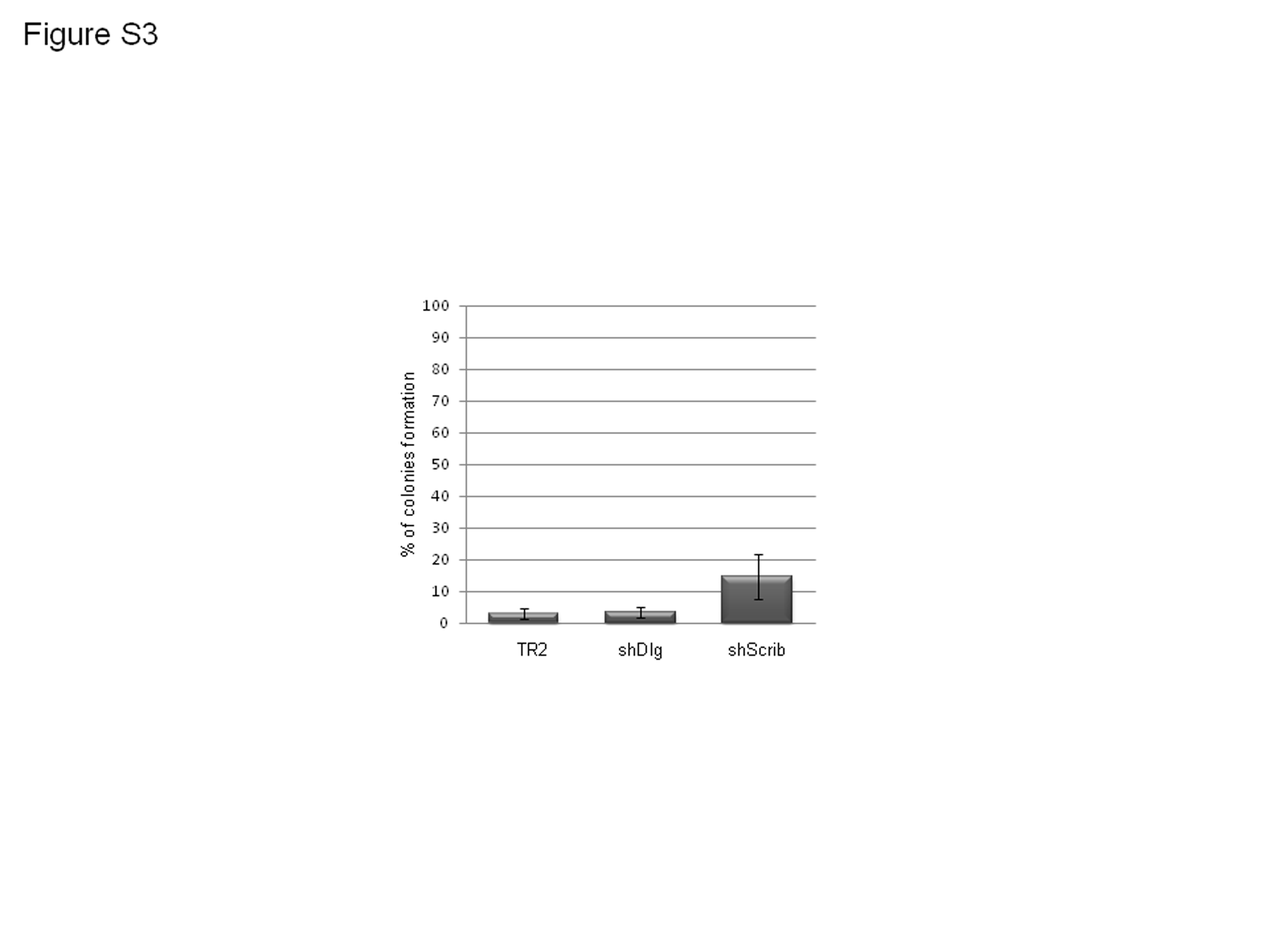

Supplement: Figure S3 — Loss of hScrib confers weak growth in soft agar. Cells were resuspended in medium containing 0.5% agar and allowed to grow for 10 days. The graph shows the numbers of colonies as a percentage of the total number of cells. Note the very weak increase in the capacity of the hScrib depleted cells to grow in soft agar. (TIF) [file pone.0040279.s003.tif]

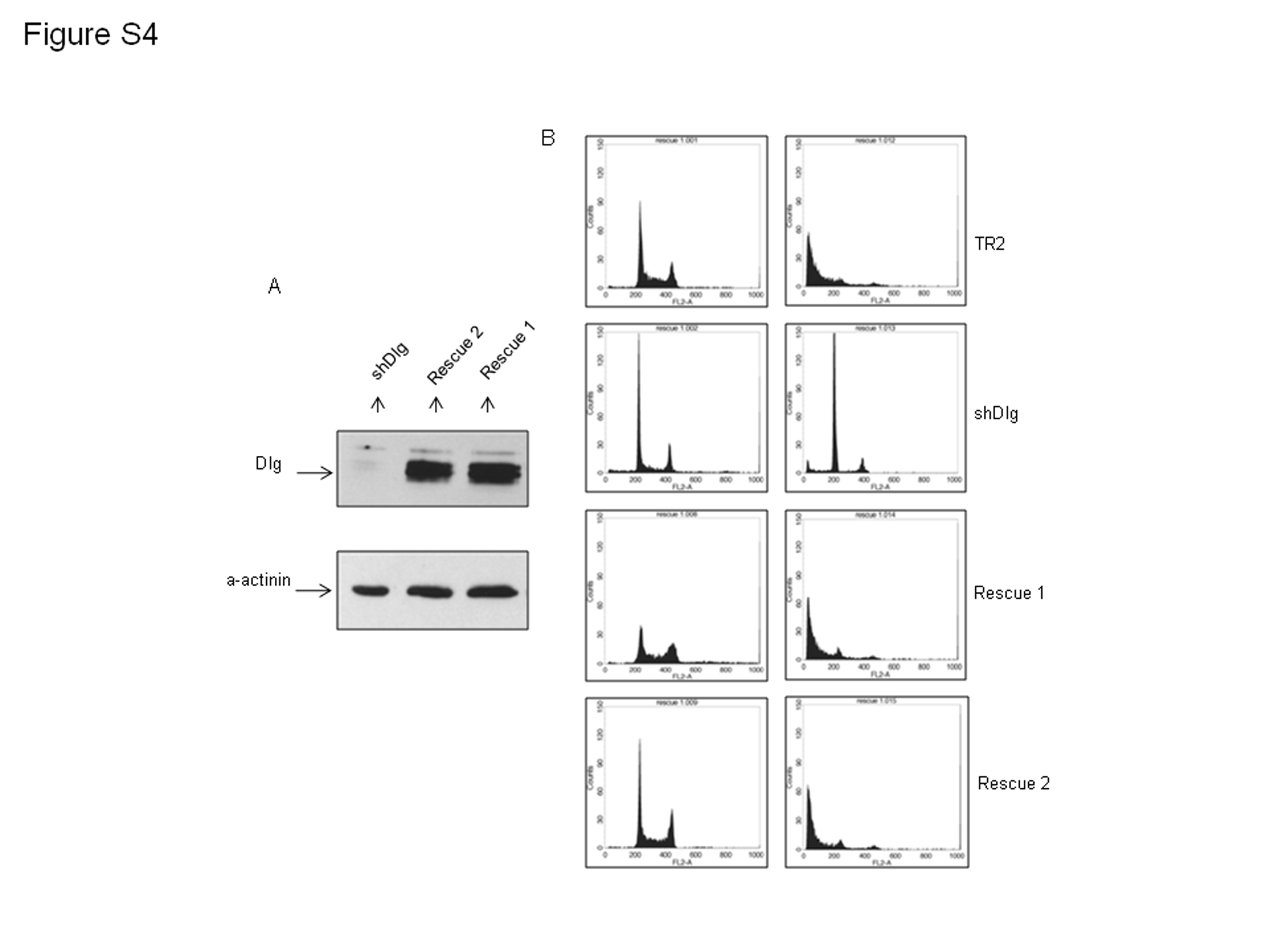

Supplement: Figure S4 — Restoration of Dlg expression restores sensitivity to anoikis. hDlg1 depleted cells and cells re-expressing rat Dlg (Panel A) were plated on poly-HEMA coated dishes. After 24 h the cells were harvested and stained with Propidium Iodide, and the cell cycle distribution of two representative clones was ascertained by flow cytometry. (TIF) [file pone.0040279.s004.tif]
